# Explainable machine learning reveals the role of taste-related genetic variants in body mass index: a nutrigenetic perspective

**DOI:** 10.3389/fnut.2026.1869879

**Published:** 2026-07-24

**Authors:** Gulsen Meral, Neval Burkay, Ahmet Avci, Merve Ozkaya, Bader Beyler, Esma Gokcen Alper Acar, Ruya Atesli, Rabia Eser, Muhammed Yunus Alp, Berna Uslu Coskun

**Affiliations:** 1 Epigenetic Coaching, London, United Kingdom; 2 Department of Pediatrics, Faculty of Medicine, Istanbul Nisantasi University, Istanbul, Türkiye; 3 Department of Biostatistics, Cerrahpaşa Faculty of Medicine, Istanbul University, Istanbul, Türkiye; 4 Department of Molecular Medicine, Institute of Health Sciences, TOBB Economics and Technology University, Ankara, Türkiye; 5 Department of Pediatrics, Acibadem Kent Hospital, Izmir, Türkiye; 6 GETAT Unit, Pediatric Health and Diseases Clinic, Aydin, Türkiye; 7 Genoks Genetic Diagnosis Center, Ankara, Türkiye; 8 Professor of Otorhinolaryngology (ENT), Istanbul, Türkiye; 9 GMC-registered Medical Practitioner, London, United Kingdom

**Keywords:** body mass index, FGF21, obesity, shap, TAS1R2, TAS1R3, TAS2R38

## Abstract

**Background:**

Taste perception–related genetic variants may influence dietary behavior and energy balance; however, their relationship with body mass index (BMI) remains unclear. This study aimed to investigate the association between taste-related genetic variants and BMI using both statistical analysis and an explainable machine learning approach.

**Methods:**

This retrospective observational study included 200 individuals aged 5–60 years with available genotype and BMI data. Variants in TAS2R38, TAS1R2, TAS1R3, and FGF21 were analyzed. Associations with BMI categories were evaluated using chi-square tests. A Random Forest classification model was developed, and SHAP (SHapley Additive exPlanations) values were used to quantify the contribution of each variant and explore allele-dose effects.

**Results:**

No significant associations were found between genetic variants and BMI categories in adults (*p* > 0.05). In children, rs35744813 showed a significant inverse association with BMI (*p* = 0.011). SHAP analysis revealed that TAS2R38 variants (rs10246939 and rs1726866) and FGF21 rs838133 demonstrated the highest relative contributions to BMI classification. Allele-dose patterns suggested differences in model attribution across genotype groups.

**Conclusion:**

Taste-related genetic variants did not demonstrate independent associations with BMI classification in the present study. However, explainable machine learning approaches identified heterogeneous model contributions across genetic variants, suggesting potential differences in genotype-related patterns within a multifactorial framework. These findings may contribute to future research exploring gene–phenotype relationships and precision nutrition approaches.

## Introduction

With globalization, especially in middle- and low-income countries, changes in dietary habits have caused a notable rise in nutrition-related non-communicable diseases (NCDs). The increased intake of processed foods high in sugar, salt, and saturated fats is a key factor in metabolic disorders such as cardiovascular diseases, type 2 diabetes, and obesity. Obesity remains a major and growing global public health issue, driven by complex interactions between dietary patterns, lifestyle factors, and biological susceptibility. In this context, understanding the biological and genetic factors that influence food preferences and eating behaviors is essential for developing effective public health strategies and personalized nutritional interventions (1, 2).

Taste perception is a key factor in food choices and works through specialized receptors that detect basic taste modalities, including bitter, sweet, umami, sour, and salty. Single-nucleotide variants (SNVs) in genes encoding taste receptors can change how sensitive a person is to certain tastes, influencing their food preferences and intake. The interaction between genetic differences and environmental factors greatly influences individual eating behaviors (1, 3).

Polymorphisms in the Taste Receptor Type 2 Member 38 (TAS2R38) gene, which encodes the bitter taste receptor, and in the Taste Receptor Type 1 Member 2 (TAS1R2) and Taste Receptor Type 1 Member 3 (TAS1R3) genes, which encode sweet taste receptor subunits, have been linked to individual differences in taste sensitivity. Variants rs713598, rs1726866, and rs10246939 in TAS2R38 are known to affect sensitivity to bitter compounds and may influence vegetable consumption habits (3–5). Similarly, the polymorphism rs35874116 in TAS1R2 has been shown to impact sweet taste perception and sugar intake. Furthermore, these effects seem more significant in individuals with a higher body mass index (BMI) and could also affect dietary responses. Variants in TAS1R3 (rs307355 and rs35744813) are also thought to influence sweet taste perception, which might relate to energy balance (5–8).

Genetic variations in taste receptors have been shown to influence not only oral perception but also metabolic regulation via the gastrointestinal system and gut-brain axis. Taste receptors are also present in the gastrointestinal system, where they affect incretin secretion, glucose balance, and energy regulation (9, 10). This indicates that genetic differences related to the sense of taste may have long-term impacts on body weight and metabolic health.

Obesity is a complex disease caused by both environmental and genetic factors. Besides the balance between energy intake and expenditure, genetic predisposition significantly influences individual susceptibility. In this context, not only taste receptor genes but also gene variants that control energy metabolism and macronutrient preferences have been linked to obesity (6, 11). Among these, Fibroblast Growth Factor 21 (FGF21), a liver-produced hormone involved in energy regulation, has attracted particular interest due to its role in controlling carbohydrate and sugar intake, appetite, and insulin sensitivity (12). Variants in the FGF21 gene, such as rs838133, have been associated with sugar intake and dietary choices (13, 14).

Although increasing evidence links taste receptor gene polymorphisms to food preferences, their direct connection with body mass index (BMI) remains unclear. Current studies are limited and yield inconsistent results. Therefore, exploring the phenotypic effects of these genetic variants, especially through retrospective datasets, may provide more insight into how taste perception and metabolic regulation influence obesity risk.

Obesity remains a significant and increasing global public health challenge. After the Human Genome Project, advancements in genomics have made it possible to predict disease risk based on genetic differences. When combined with clinical history and phenotypic data, this approach underpins precision medicine, which seeks not only to treat diseases but also to forecast risks, implement preventive measures, and create personalized interventions (2). From a healthy aging and longevity standpoint, early recognition of individual dietary habits, prompt identification of risk behaviors, and customized lifestyle planning are crucial. Personalized nutrition and tailored lifestyle strategies are therefore key elements of longevity-focused healthcare.

In this context, the present retrospective study aimed to explore the connection between BMI and genetic variants in TAS2R38 (rs10246939, rs1726866, rs713598), TAS1R3 (rs35744813), TAS1R2 (rs35874116), and FGF21 (rs838133) in a cohort of 200 individuals. This approach aims to strengthen the nutrigenetic framework linking genotype to phenotype and to provide a scientific basis for personalized nutritional strategies in preventing metabolic diseases.

## Methods

### Study design and ethics

This retrospective, observational, archive-based study was designed to evaluate the relationship between taste perception–related genetic polymorphisms and BMI, using statistical and machine learning methods applied to archived genotype and BMI data. This research is based on a review of records previously obtained during routine procedures and does not involve any intervention, new sample collection, or contact with participants. The study was conducted according to the principles of the Declaration of Helsinki, and the relevant ethics committee processes were completed. The study was reviewed at the ethics committee meeting held on December 18, 2025, numbered 2025-09, and was approved by the Ethics Committee of Istanbul Nisantasi University Faculty of Health Sciences.

### Research universe and sample

The study population included individuals aged 5–60 years whose genetic test results related to taste were recorded in the institutional archive. The sample was selected from individuals with complete genetic data and BMI records in the archive within a specific timeframe.

### Participants and study design

This retrospective observational study used archived records from individuals aged 5–60 years who had previously undergone genetic testing. Eligibility was determined based on predefined inclusion criteria. The study included 200 participants with complete genotype and BMI data. Due to the retrospective archive-based design, no prior sample size calculation was performed; instead, all eligible records with complete data available during the study period were included. To reduce information bias, only records with complete genotype and BMI data were analyzed, and all data were reviewed in anonymized form according to predefined eligibility criteria. Participants were selected from individuals who had previously undergone genetic testing for personal health assessment purposes unrelated to this study sample was drawn from a general population. The genetic data used herein was therefore collected prior to and independent of the current research.

### Data sources and variables

#### Genetic variables

Genetic data were obtained from archived genotyping reports based on previously collected salivary samples. No new laboratory analyses were conducted in this study. The following polymorphisms were assessed.

- Bitter taste receptor-related gene polymorphisms: TAS2R38 rs10246939, rs1726866, rs713598
- Sweet taste receptor-related gene polymorphisms: TAS1R3 rs35744813; TAS1R2 rs35874116
- Metabolism- and sweet preference-related gene polymorphism: FGF21 rs838133

To avoid implying direct causality for obesity, these alleles are called effect alleles. For modeling, genotypes were coded as 0, 1, or 2 copies of the effect allele in an additive manner. Each SNP was analyzed independently to assess its individual contribution to BMI classification. This single-SNP approach was intentionally chosen to avoid assumptions about the relative weight of each variant in the absence of prior evidence supporting a specific weighting scheme in this population. Composite or weighted scoring approaches may be explored in future studies and have been acknowledged in the limitations section.

#### Genetic analysis

Genomic DNA was extracted from buccal swab samples using the phenol-chloroform method. The concentration and purity of the DNA were measured with a Nanodrop 1,000 spectrophotometer (Thermo Scientific, Wilmington, DE, USA), and all samples were adjusted to a final concentration of 50 ng/μL. Genotyping was conducted using a custom Infinium HTS iSelect microarray platform (Illumina, San Diego, CA, USA), following the manufacturer's protocol (15).

#### Phenotypic and clinical variables

Phenotypic data have only been extracted from the existing files.

- Height (cm) and body weight (kg)
- Body Mass Index (BMI; kg/m^2^)

Anthropometry, BMI classification height (cm), and body weight (kg) were obtained from archived records. Body mass index (BMI) was calculated as weight (kg) divided by height (m) squared. Adults and children were analyzed separately to use age-appropriate BMI classification systems. For adults (≥18 years), BMI categories were defined based on World Health Organization (WHO) cutoff points: underweight (< 18.5 kg/m^2^), normal weight (18.5–24.9 kg/m^2^), overweight (25.0–29.9 kg/m^2^), and obesity (≥30.0 kg/m^2^), with obesity classes reported when relevant (Class I: 30.0–34.9; Class II: 35.0–39.9; Class III: ≥40.0 kg/m^2^) (16).

For children and adolescents (5–17 years old), BMI-for-age was classified using the WHO 2007 growth reference based on BMI-for-age z-scores (BAZ) as follows: thinness (< -2 SD), normal weight (−2 SD to +1 SD), overweight (>+1 SD), and obesity (>+2 SD). Severe thinness and obesity were defined as BAZ < -3 SD and BAZ >+3 SD, respectively (17).

#### Inclusion and exclusion criteria

##### Inclusion criteria

- The genotypic data for the identified SNVs can be found in the archive.
- Having height and weight data that enables BMI calculation,
- The records are adequate and consistent for analysis.

##### Exclusion criteria

- Missing, unreadable, or incorrect genetic data records.
- Records lacking BMI data,
- Records that are unsuitable for the anonymization or coding process or have compromised data integrity.

### Data extraction, anonymization, and data security

The archive review was carried out by authorized researchers using the institution's information system and patient files. Personally identifiable information was removed before transferring the analysis file, and a unique code was assigned to each record. The dataset was stored in an encrypted digital environment and accessible only to the research team. The study was conducted in accordance with data processing principles compliant with the Personal Data Protection Law.

### Statistical analysis

The statistical analysis of the data collected in this study was performed using IBM SPSS Statistics 2023 and RStudio (R version 4.2.3). Descriptive statistics and graphical visualizations of demographic characteristics were generated within both software environments. During data pre-processing and analysis, the R environment was utilized together with the readxl, janitor, dplyr, and tidyr packages.

The demographic characteristics of the participants, including age, height, body weight, and BMI, were summarized using descriptive statistics. Continuous variables are presented as mean ± standard deviation and median (minimum–maximum) values, whereas categorical variables are expressed as frequencies and percentages. Box plots were additionally used to visually inspect data distributions and identify potential outliers. No extreme outliers requiring exclusion were identified; therefore, all observations were retained in the analysis.

Genotype quality control procedures were performed prior to statistical analyses. Genotyping completeness was evaluated using call rates and missing genotype frequencies. Minor allele frequencies (MAF) and Hardy–Weinberg equilibrium (HWE) were assessed for all investigated SNPs using exact tests. In accordance with this suggestion, we have now included the essential genotyping quality-control (QC) metrics in the Materials and Methods section.

To evaluate the association between taste perception–related genetic variants and BMI categories, exact chi-square tests with Monte Carlo simulation were applied due to low expected frequencies across several genotype categories.

Additionally, age- and sex-adjusted multinomial logistic regression analyses were performed to evaluate whether genotype distributions were independently associated with BMI categories while accounting for potential demographic confounding effects. Statistical inference from adjusted models was primarily based on overall model significance because sparse genotype frequencies produced unstable individual parameter estimates.

A Random Forest–based machine learning approach was applied as an exploratory modeling technique to evaluate the relative contribution of genetic variants to BMI classification. For machine learning analyses, BMI was modeled as a binary outcome, and participants were classified as overweight/obese vs. non-overweight/non-obese. This binary classification framework was used for Random Forest model development and subsequent SHAP interpretation. After model construction, SHAP (SHapley Additive exPlanations) analysis was used to interpret model prediction behavior and quantify the relative contribution of variables to model output. Effect allele counts (0, 1, and 2) for SNVs were included as numerical variables in the model, and genotype-specific SHAP distributions were evaluated to explore allele-dose patterns. SHAP values were interpreted as indicators of model contribution and were not considered evidence of biological causality or independent genetic risk.

In all statistical analyses, *p* < 0.05 was considered statistically significant.

## Results

### Demographic findings

A total of 200 individuals, including 100 men and 100 women, participated in this study. The mean age of the male participants was 33.1 ± 17.7 years, with a median age of 35.5 years (min-max: 5-60). Their average height was 167 ± 20.4 cm (median: 175 cm, min-max: 110–190 cm), and the average body weight was 73.6 ± 26.7 kg (median: 80 kg, min-max: 16–135 kg). The female participants had a mean age of 39.1 ± 14.1 years, with a median age of 42 years (min-max: 5–60). Their average height was 162 ± 10.2 cm (median: 165 cm, min-max: 123–181 cm), and their mean body weight was 62.7 ± 14.5 kg (median: 62 kg, min-max: 22–98 kg) (Table 1).

**Table 1:** Demographic characteristics of participants (height, weight, and age).

Gender	Variable	Mean ±SD	Median	Min–Max
Male (*n* = 100)	Age	33.1 ± 17.7	35.5	5–60
	Height (cm)	167 ± 20.4	175	110–190
	Weight (kg)	73.6 ± 26.7	80	16–135
Female (*n* = 100)	Age	39.1 ± 14.1	42	5–60
	Height (cm)	162 ± 10.2	165	123–181
	Weight (kg)	62.7 ± 14.5	62	22–98

### Findings of child and adult BMI

The study population consisted of 200 participants, including 160 adults (71 males and 89 females; ≥18 years) and 40 children (29 boys and 11 girls; < 18 years). When examining BMI values in adults, the average BMI for males was 27.5 ± 4.16 kg/m^2^, with a median of 26.9 kg/m^2^ (min-max: 19.7–41.7). In females, the average BMI was 24.1 ± 4.45 kg/m^2^, with a median of 23.1 kg/m^2^ (min-max: 17.1–40.8). These results show that adult males tend to have higher BMI values than females.

When assessing the BMI SDS values in the children group, the mean BMI SDS for boys was 0.321 ± 1.57, with a median of 0.31 (min-max: −3.08 to 3.42); for girls, the mean BMI SDS was 0.669 ± 1.38, with a median of 0.74 (min-max: −1.36 to 2.46) (Table 2).

**Table 2:** Body mass index for adults and children.

BMI	Gender	*N*	Mean ±SD	Median	Min-Max
Adult	Male	71	27.5 ± 4.16	26.9	19.7–41.7
	Female	89	24.1 ± 4.45	23.1	17.1–40.8
Child	Male	29	0.321 ± 1.57	0.31	−3.08–3.42
	Female	11	0.669 ± 1.38	0.74	−1.36–2.46

When examining the distribution of children by BMI classification, 52.5% of the participants were of normal weight, 20.0% were overweight, and 17.5% were obese. The rates of severe thinness and morbid obesity were each found to be 2.5% (Table 3).

**Table 3:** Body mass index percentile classifications in children.

Classification	Frequency	Percentage
Severe thinness	1	%2.5
Thinness	2	%5.0
Normal	21	%52.5
Overweight	8	%20.0
Obese	7	%17.5
Severely obese	1	%2.5

When BMI categories were assessed in adults, approximately half of the participants (48.8%) were classified as normal weight, 32.5% as overweight, 12.5% as class I obese, 1.9% as class II obese, and 1.3% as morbidly obese (Table 4).

**Table 4:** Body mass index percentile classification for adults.

Classification	Frequency	Percentage
Thinness	5	%3.1
Normal weight	78	%48.8
Overweight	52	%32.5
Class I obesity	20	%12.5
Class II obesity (Severe obesity)	3	%1.9
Class III obesity (Morbid obesity)	2	%1.3

### Distribution of BMI categories based on gene polymorphisms in bitter and sweet taste receptors among adults

When examining the distribution of gene polymorphisms in bitter and sweet taste receptors across BMI categories in adults, genotype frequencies appeared to be broadly comparable among underweight, normal weight, overweight, and obesity groups. For the bitter taste receptor–related polymorphisms, no statistically significant differences in BMI classification were observed across rs10246939, rs1726866, and rs713598 genotype groups (*p* = 0.871, *p* = 0.782, and *p* = 0.784, respectively). Likewise, no statistically significant associations were identified between BMI categories and the evaluated sweet taste receptor and metabolism-related variants, including rs35744813, rs35874116, and rs838133 (all *p* > 0.05) (Table 5). These findings indicate that the investigated SNPs were not individually associated with BMI category distribution in the adult subgroup.

**Table 5:** Chi-Square test of the association between bitter and sweet taste receptor gene polymorphisms and body mass index categories in adults.

Gene Category	Gene	SNP	Effect Allele^*^	Genotype	Thinness (*N*)	Normal (*N*)	Overweight (*N*)	Class I (*N*)	Class II (*N*)	Class III (*N*)	*p*
Bitter taste receptor related gene polymorphisms	TAS2R38 TAS2R38	rs10246939 rs1726866	C	TT	1	17	17	5	1	1	0.871 0.782
				CT	3	39	22	8	2	0	
				CC	1	22	13	7	0	1	
			G	AA	1	17	17	5	1	1	
				AG	3	39	22	7	2	0	
				GG	1	22	13	8	0	1	
	TAS2R38	rs713598	G	CC	1	21	13	7	0	1	0.784
				CG	3	36	21	5	2	0	
				GG	1	21	18	8	1	1	
Sweet taste receptor related gene polymorphisms	TAS1R3	rs35744813	T	CC	4	65	40	16	3	2	0.973 0.398
				CT	1	12	11	4	0	0	
				TT	0	1	1	0	0	0	
	TAS1R2	rs35874116	C	TT	3	39	25	9	2	0	
				CT	1	37	26	9	1	2	
				CC	1	2	1	2	0	0	
Metabolism- and sweet preference related gene polymorphism	FGF21	rs838133	A	GG	1	23	16	2	1	0	0.521
				AG	3	42	28	17	2	2	
				AA	1	13	8	1	0	0	

^*^Effect allele was defined separately for each SNP according to previously published literature. All alleles are presented in forward orientation. Associations were evaluated using exact chi-square tests with Monte Carlo simulation due to sparse genotype frequencies.

Adults and children were analyzed separately to use age-appropriate BMI classification systems. For adults (≥18 years), BMI categories were defined based on World Health Organization (WHO) cutoff points: underweight (< 18.5 kg/m^2^), normal weight (18.5–24.9 kg/m^2^), overweight (25.0–29.9 kg/m^2^), and obesity (≥30.0 kg/m^2^), with obesity classes reported when relevant (Class I: 30.0–34.9; Class II: 35.0–39.9; Class III: ≥40.0 kg/m^2^) (16).

For children and adolescents (5–17 years old), BMI-for-age was classified using the WHO 2007 growth reference based on BMI-for-age z-scores (BAZ) as follows: thinness (< -2 SD), normal weight (−2 SD to +1 SD), overweight (>+1 SD), and obesity (>+2 SD). Severe thinness and obesity were defined as BAZ < -3 SD and BAZ >+3 SD, respectively (17).

In children, no statistically significant associations were identified between BMI categories and the bitter taste receptor–related polymorphisms rs10246939, rs1726866, and rs713598 (*p* = 0.291, *p* = 0.291, and *p* = 0.546, respectively). Likewise, no statistically significant associations were observed between BMI categories and the evaluated sweet taste receptor and metabolism-related variants, including rs35874116 and rs838133 (all *p* > 0.05).

A statistically significant difference in BMI category distribution was observed for the rs35744813 polymorphism (*p* = 0.011). However, this finding should be interpreted cautiously because genotype frequencies were sparse and some genotype groups contained a limited number of observations. For rs35744813, most participants were concentrated in the CC genotype group across normal weight, overweight, and obesity categories, whereas CT and TT genotypes were represented by relatively few individuals (Table 6).

**Table 6:** Chi-Square test of the association between bitter and sweet taste receptor gene polymorphisms and body mass index categories in children.

Gene Category	Gene	SNP	Effect allele^*^	Genotype	Thinness (*N*)	Normal (*N*)	Overweight (*N*)	Class I (*N*)	Class II (*N*)	Class III (*N*)	*p*
Bitter taste receptor related gene polymorphisms	TAS2R38	rs10246939	C	TT	0	1	10	1	1	0	0.291 0.291 0.546
				CT	1	1	5	6	5	1	
				CC	0	0	6	1	1	0	
	TAS2R38	rs1726866	G	AA	0	1	10	1	1	0	
				AG	1	1	5	6	5	1	
				GG	0	0	6	1	1	0	
	TAS2R38	rs713598	G	CC	0	0	4	1	1	0	
				CG	1	1	7	6	5	0	
				GG	0	1	10	1	1	1	
Sweet taste receptor related gene polymorphisms	TAS1R3	rs35744813	T	CC	0	1	18	8	7	1	0.011 0.731
				CT	0	1	3	0	0	0	
				TT	1	0	0	0	0	0	
	TAS1R2	rs35874116	C	TT	1	1	8	6	4	0	
				CT	0	1	11	2	2	1	
				CC	0	0	2	0	1	0	
Metabolism- and sweet preference related gene polymorphism	FGF21	rs838133	A	GG	0	0	4	1	1	0	0.533
				AG	0	2	10	7	4	1	
				AA	1	0	7	0	2	0	

^*^Effect allele was defined separately for each SNP according to previously published literature. All alleles are presented in forward orientation. Associations were evaluated using exact chi-square tests with Monte Carlo simulation due to sparse genotype frequencies.

### Supplementary age- and sex-adjusted multinomial logistic regression results

Additional multinomial logistic regression analyses adjusted for age and sex were performed to evaluate whether the investigated SNPs were independently associated with BMI categories in adults. The adjusted analyses showed no statistically significant overall associations between BMI categories and the evaluated genetic variants, including rs10246939 (*p* = 0.74), rs1726866 (*p* = 0.82), rs713598 (*p* = 0.61), rs35744813 (*p* = 0.48), rs35874116 (*p* = 0.39), and rs838133 (*p* = 0.55). These findings remained consistent with the primary chi-square analyses and suggest that the investigated SNPs were not independently associated with BMI classification after accounting for age and sex.

### Random forest model performance

Detailed Random Forest performance metrics for BMI classification are presented in Supplementary Table S4. While the model demonstrated moderate discrimination in the training dataset (AUC = 0.758), its performance declined in the independent test set and cross-validation analyses (test AUC = 0.534; CV AUC = 0.461), suggesting limited generalizability. Additional evaluation metrics, including Accuracy, Sensitivity, Specificity, Precision, F1-score, and Kappa, further indicated weak classification performance beyond chance. Accordingly, SHAP findings were interpreted as exploratory indicators of relative feature contribution patterns rather than robust predictive biomarkers.

#### SHAP analysis

SHAP analysis was used to assess each SNP's contribution to the Random Forest model classification decisions. In this framework, positive SHAP values indicate that the feature contributed toward classification into the overweight/obese class, whereas negative SHAP values indicate contribution toward classification into the non-overweight/non-obese class.

Among bitter taste receptor–related polymorphisms, rs10246939 (C allele) and rs1726866 (G allele) demonstrated the highest mean SHAP contributions to the model output, with mean SHAP values of 0.33 and 0.25, respectively. The rs713598 (G allele) variant showed a relatively lower contribution (mean SHAP = 0.15). Among sweet taste receptor–related polymorphisms, rs35744813 (T allele) and rs35874116 (C allele) demonstrated comparatively limited contributions, with mean SHAP values of 0.11 and 0.14, respectively. The metabolism- and sweet preference–related variant rs838133 (A allele) showed a relatively higher contribution (mean SHAP = 0.21) compared with the remaining sweet receptor variants (Table 7).

**Table 7:** Mean absolute SHAP Values for SNP contributions in the random forest model.

Taste receptor genes	SNP	Effect allele^*^	Mean SHAP
TAS2R38	rs10246939	C	0.3283
TAS2R38	rs1726866	G	0.2524
TAS2R38	rs713598	G	0.1486
TAS2R38	rs10246939	C	0.0098
TAS2R38	rs1726866	G	0.1037
TAS1R3	rs35744813	T	0.1185
TAS1R2	rs35874116	C	0.1422
TAS2R38	rs713598	G	0.0450
FGF21	rs838133	A	0.2138

^*^Effect Allele; allele associated with increased sweet taste preference. All alleles are presented in the forward orientation.

**Figure 1:**
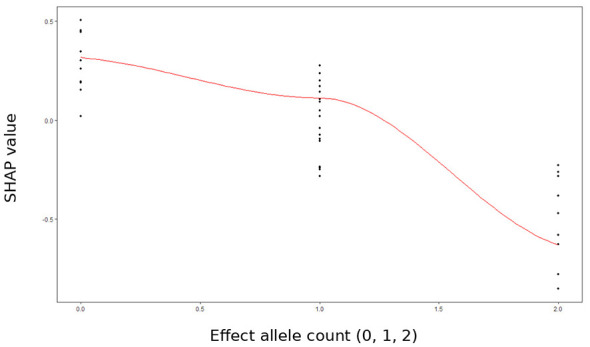
SHAP values for the C allele of SNV rs10246939. Each dot represents one individual participant. The number of observations at each allele count reflects the sample size of the corresponding genotype group.

Figure 1 presents SHAP values for the C allele of the rs10246939 SNV. The x-axis represents effect allele count (0, 1, and 2), and the y-axis represents SHAP values for individual observations. Positive SHAP values indicate greater contribution toward classification into the overweight/obese class, whereas negative SHAP values indicate contribution toward classification into the non-overweight/non-obese class.

For the bitter taste receptor–related rs10246939 SNV, heterozygous observations (one effect allele) showed relatively limited SHAP variation, whereas homozygous observations demonstrated lower SHAP values across several observations, indicating differences in model attribution patterns across genotype groups (Figure 1).

**Figure 2:**
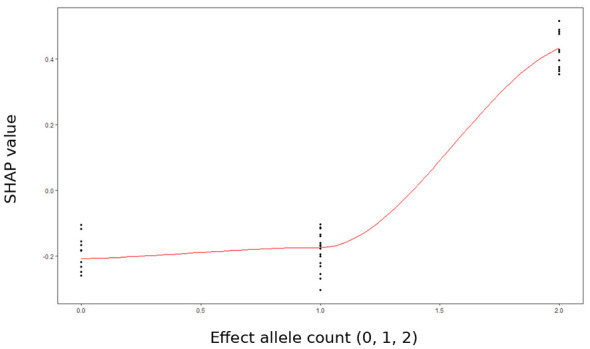
SHAP values for the G allele of SNV rs1726866. Each dot represents one individual participant. The number of observations at each allele count reflects the sample size of the corresponding genotype group.

Figure 2 presents SHAP values according to effect allele count (0, 1, and 2) for the investigated SNV. The x-axis represents allele count, whereas the y-axis displays SHAP values for individual observations. Lower allele counts (0 and 1) were generally associated with negative SHAP values, whereas observations with two effect alleles tended to show higher and more positive SHAP values. The smoothed trend line indicated an increasing pattern of SHAP values across allele counts, suggesting differences in model attribution across genotype groups (Figure 2).

**Figure 3:**
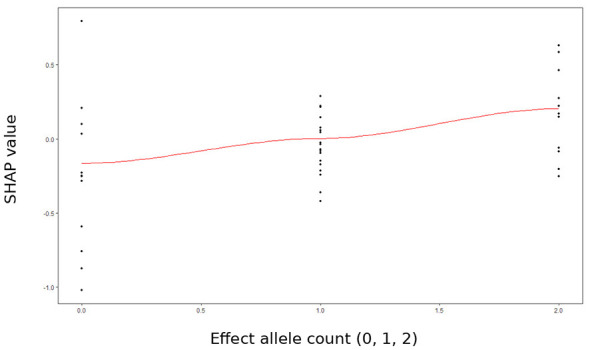
SHAP values for the A allele of SNV rs838133. Each dot represents one individual participant. The number of observations at each allele count reflects the sample size of the corresponding genotype group.

In Figure 3, SHAP values for the sweet taste–related rs838133 SNV are presented according to effect allele count (0, 1, and 2). The SHAP value represents the relative contribution of each genotype to the Random Forest model's output. As shown in Figure 3, observations with higher effect allele counts tended to demonstrate slightly higher SHAP values, although substantial variability was observed within genotype groups. The smoothed trend line suggested modest in model attribution across allele counts (Figure 3).

## Discussion

In this retrospective study, we aimed to contribute to the literature by examining the relationship between taste perception-related genetic polymorphisms and BMI, not only through traditional univariate statistical comparisons but also with a machine-learning-based explainability approach. The study analyzed data from 200 individuals aged 5–60 years. Although no statistically significant association was found between the examined variants and BMI categories in the adult group, a significant association was observed between BMI categories and the TAS1R3 rs35744813 variant in the child group (Tables 5, 6). Additionally, SHAP analysis indicated relatively higher contribution patterns for TAS2R38 variants (rs10246939 and rs1726866) and the FGF21 rs838133 variant within the Random Forest model (Figures 1–3). In our study, no significant differences were found in BMI classifications among the genotype groups for the TAS2R38 polymorphisms in the bitter taste receptor-related gene polymorphisms (rs10246939, rs1726866, and rs713598; *p* = 0.871, *p* = 0.782, and *p* = 0.784, respectively). Additionally, carrying the effect allele did not appear to significantly affect weight status (Table 5). However, the relatively higher SHAP contributions observed for TAS2R38 variants should not be interpreted as evidence of biological importance. Instead, these findings may reflect model attribution patterns and could serve as exploratory observations for future studies investigating taste perception and adiposity (18). TAS2R38 is one of the most extensively studied genes in bitter taste perception, and its variants have been associated with vegetable consumption, bitter taste tolerance, and food preference behaviors (4, 5). Some research suggests an association between TAS2R38 variants and obesity risk.

In this study, TAS2R38 rs10246939 and rs1726866 polymorphisms demonstrated relatively higher SHAP contributions among bitter taste receptor–related variants, whereas rs713598 showed a comparatively lower contribution (Table 7). These findings indicate differences in model attribution across genotype groups rather than direct biological effects. For TAS2R38 rs10246939, heterozygous observations showed relatively limited variation in SHAP values, whereas homozygous observations demonstrated lower SHAP values across several observations (Figure 1). For TAS2R38 rs1726866, higher effect allele counts tended to correspond to more positive SHAP values within the model output (Figure 2). These findings suggest variability in model contribution patterns across genotype groups and support the use of explainable machine learning approaches for exploring genotype–phenotype relationships. Similarly, for the polymorphisms evaluated in the sweet taste receptor-related gene polymorphisms (rs10246939, rs1726866, rs35744813, rs35874116, rs713598, and rs838133), genotype distributions did not differ significantly across BMI categories (all *p* > 0.05) (Table 5). However, the relatively higher mean SHAP contribution observed for the FGF21 rs838133 variant in metabolism- and sweet preference–related pathways is noteworthy (Figure 3; Table 7). Although FGF21 is not a classical taste receptor gene, previous studies have associated this pathway with energy homeostasis, carbohydrate preference, and metabolic regulation (13, 14). In contrast, the relatively limited SHAP contributions observed for TAS1R2 rs35874116 and TAS1R3 rs35744813 may indicate differences in model attribution patterns across sweet taste–related genetic variants. Findings indicating that reduced taste sensitivity is linked to higher adiposity are particularly significant. In older adults with metabolic syndrome, lower overall taste scores have been reported to be inversely related to body weight, BMI, and waist circumference (19). Another study That shown that the TAS1R2 rs35874116 CC genotype is associated with greater sensitivity to low sucrose concentrations, whereas TT carriers may exhibit reduced sweet taste sensitivity. Similarly, the TAS1R3 rs35744813 T allele has been linked to decreased sweet taste sensitivity due to reduced promoter activity and lower receptor expression. These genetic differences may contribute to inter-individual variability in sweet taste perception, dietary preferences, sugar intake, and potentially BMI (20, 21). Furthermore, twin studies have shown that sweet taste perception during adolescence may predict BMI in early adulthood and that these traits may share a common genetic basis, providing additional evidence for a biological connection between taste phenotype and adiposity (22). However, the link between taste receptor polymorphisms and BMI has not always been consistent. For example, some studies suggest that the effects of certain sweet receptor-related variants can vary depending on BMI. While these variants are associated with decreased sweet sensitivity and increased sugar intake in overweight individuals, they may have the opposite effect in lean individuals (23). Research in the Turkish population also found no connection between TAS1R2 variants and obesity (24, 25). Likewise, in our study, no significant link was found between the TAS1R2 gene rs35874116 polymorphism and BMI. This consistency may also highlight why obesity is influenced by complex gene-environment interactions; in other words, obesity is a multifactorial condition. Therefore, while genetic factors that affect taste perception can influence food preferences, environmental factors such as diet, food availability, physical activity, and cultural influences also contribute to BMI. Consequently, the significant variation in distribution can be understood within this context. In addition, the absence of statistically significant associations in the adult cohort should not necessarily be interpreted as evidence of the absence of genetic effects. The investigated variants are expected to exert relatively modest effects on complex phenotypes such as BMI, and these effects may have been difficult to detect given the available sample size. Furthermore, differences in ethnicity, dietary habits, environmental exposures, and population characteristics across studies may also contribute to the inconsistent findings reported in the literature.

In the child group, a statistically significant inverse association was observed between BMI categories and the TAS1R3 rs35744813 polymorphism among sweet taste receptor–related variants (*p* = 0.011) (Table 6). This finding may suggest that genotype–BMI associations could differ across age groups and warrants further investigation in larger pediatric populations. Previous studies have reported associations between taste receptor SNVs and taste-related phenotypes in children and young adults and suggested that such variation may contribute to differences in food preferences (26, 27). Interestingly, the observed inverse association was not consistent with expectations regarding obesity prevalence. Previous studies have also reported no significant association between this variant and BMI (8, 20). Although this variant has been discussed in relation to taste-related phenotypes, its relationship with complex outcomes such as obesity remains unclear. In the child group, a statistically significant association was observed between BMI categories and the TAS1R3 rs35744813 polymorphism among sweet taste receptor–related variants (*p* = 0.011) (Table 6). However, this finding should be interpreted cautiously because the pediatric subgroup included only 40 participants and several genotype categories contained a limited number of observations. Although exact chi-square testing with Monte Carlo simulation was applied to account for sparse cell frequencies, the observed finding should be interpreted carefully and requires confirmation in larger pediatric cohorts before drawing firm conclusions regarding this association. Although formal multiple-comparison correction was not applied because the analyses were hypothesis-driven and focused on predefined candidate SNPs, we acknowledge that the statistically significant association observed for TAS1R3 rs35744813 in the pediatric subgroup may not remain statistically significant after conservative correction methods such as Bonferroni adjustment. Therefore, this finding should be regarded as exploratory and requires independent replication before any firm conclusions can be drawn.

### Strengths, limitations, and future directions

The most significant strength of this study is demonstrating an inverse relationship between BMI and genotype in children. Another key strength is combining traditional univariate testing with an explainable machine learning framework. While univariate comparisons in adults did not show statistically significant genotype-BMI links, the Random Forest model with SHAP analysis uncovered that several SNVs provided discriminative information in a combined, multivariable pattern. This complementary approach is especially valuable for complex traits like taste perception and eating behavior, where individual variants may have minimal effects that only become apparent when assessed together. Future studies could be enhanced by including a polygenic risk score (PRS), which integrates the effects of multiple variants into a single measure of genetic risk for complex traits.

Several limitations must be acknowledged. First, the retrospective design prevents establishing causality. Second, residual confounding cannot be ruled out because detailed data on dietary intake, physical activity, socioeconomic factors, comorbidities, and medication use were not consistently available for adjustment. Third, BMI is an imperfect measure of adiposity because it does not account for fat distribution or body composition, potentially weakening the signals between genotype and phenotype. Lastly, subgroup analyses, especially in children, may be impacted by small cell counts for less common genotypes, resulting in reduced statistical power and unstable estimates. Additionally, two investigated variants (rs35874116 and rs838133) deviated from Hardy–Weinberg equilibrium in the overall sample (Supplementary Table S1). Given the retrospective archive-based design and non-population-based sampling framework, this deviation may reflect population heterogeneity, selection characteristics of the available records, or sampling-related variation rather than genotyping error alone. Nevertheless, these findings should be considered when interpreting the results and warrant confirmation in larger independent cohorts.

An additional important limitation is the absence of detailed dietary intake and eating behavior data. The investigated taste-related genetic variants are hypothesized to influence body weight primarily through modulation of taste perception, food preference, sugar consumption, and dietary choices rather than through direct metabolic effects. Consequently, without quantitative information on habitual dietary intake, food preferences, or eating patterns, it was not possible to evaluate the biological pathway linking genotype to BMI. Therefore, the observed lack of association between these genetic variants and BMI should not be interpreted as evidence against their potential influence on dietary behavior. Future prospective studies integrating comprehensive dietary assessments together with genetic and metabolic data are warranted to better elucidate these complex gene–behavior–phenotype relationships.

Future research should focus on larger, ethnically diverse cohorts and prospective designs with standardized phenotyping. Incorporating detailed dietary records, objective physical activity data, and body composition measures (such as waist circumference, fat mass, and visceral fat) would facilitate a more accurate evaluation of gene behavior metabolism interactions. Furthermore, multilocus strategies such as PRS and models explicitly testing gene-environment interactions may better capture the small yet cumulative effects of taste-related genetics on BMI and related metabolic outcomes. The relatively low predictive performance of the Random Forest model indicates that the investigated genetic variants alone are insufficient for accurate BMI classification. This finding further supports the multifactorial nature of obesity, in which environmental exposures, lifestyle factors, and additional genetic determinants likely contribute more substantially than the limited set of taste-related variants evaluated in the present study.

Recent studies employing SHAP-based explainable machine learning frameworks for obesity classification further support the methodological validity of the approach adopted in the present study, demonstrating that interpretable ensemble models can effectively identify key contributors to BMI-related outcomes (28). Recent advances in medical artificial intelligence demonstrate a clear evolution from conventional CNN-based models to transformer-based architectures. Gandhi et al. (29) reported that a VGG16-based framework achieved high diagnostic performance and improved interpretability in glaucoma detection. In a subsequent study, the same research group showed that a multimodal Vision Transformer (ViT)-based model integrating retinal fundus images and optical coherence tomography (OCT) scans outperformed traditional CNN architectures. These findings highlight the growing potential of transformer-based approaches for improving disease diagnosis, clinical decision support, and precision medicine applications (30).

## Conclusion

This study suggests that taste perception–related genetic variants do not demonstrate independent associations with BMI classification but may exhibit heterogeneous contribution patterns within a multifactorial framework. While conventional statistical analyses identified limited associations, explainable machine learning provided additional insight into variation in model attribution across genetic variants. These findings support further investigation of gene–phenotype relationships and may inform future research in nutrition and metabolic health. Future prospective studies incorporating lifestyle, dietary intake, and metabolic parameters are warranted to better understand these relationships.

## Data Availability

The raw data supporting the conclusions of this article will be made available by the authors, without undue reservation.
