# Supplementary material for: Explainable machine learning reveals the role of taste-related genetic variants in body mass index: a nutrigenetic perspective
Source: Front Nutr. 2026 Jul 24;13:1869879. doi: 10.3389/fnut.2026.1869879 (PMC13447141; doi:10.3389/fnut.2026.1869879)
Supplement: Supplementary file 1 [file Table_1.docx]

SUPPLEMENTARY MATERIAL

Supplementary Table S1.
Genotype Quality Control Metrics for Investigated SNPs

Genotype quality control analyses demonstrated complete genotyping success across all investigated SNPs, with a call rate of 100% and no missing genotype data. Minor allele frequencies (MAF) ranged between 0.099 and 0.494, indicating adequate allele representation for subsequent association analyses. Most evaluated variants were consistent with Hardy–Weinberg equilibrium expectations, including rs10246939 (p = 0.435), rs1726866 (p = 0.348), rs713598 (p = 0.061), and rs35744813 (p = 0.654). However, rs35874116 (p = 0.027) and rs838133 (p = 0.017) showed deviation from Hardy–Weinberg equilibrium in the overall study sample (Supplementary Table S1).

| SNP | Call Rate (%) | Missing Genotype Rate (%) | MAF | HWE p-value |
| --- | --- | --- | --- | --- |
| rs10246939 | 100 | 0 | 0.494 | 0.435 |
| rs1726866 | 100 | 0 | 0.491 | 0.348 |
| rs713598 | 100 | 0 | 0.478 | 0.061 |
| rs35744813 | 100 | 0 | 0.099 | 0.654 |
| rs35874116 | 100 | 0 | 0.272 | 0.027 |
| rs838133 | 100 | 0 | 0.435 | 0.017 |

Supplementary Table S2.
Age- and Sex-Adjusted Multinomial Logistic Regression Results

Additional multinomial logistic regression analyses adjusted for age and sex were performed to evaluate whether the investigated SNPs were independently associated with BMI categories in adults. The adjusted analyses showed no statistically significant overall associations between BMI categories and the evaluated genetic variants, including rs10246939 (p = 0.74), rs1726866 (p = 0.82), rs713598 (p = 0.61), rs35744813 (p = 0.48), rs35874116 (p = 0.39), and rs838133 (p = 0.55). These findings remained consistent with the primary chi-square analyses and suggest that the investigated SNPs were not independently associated with BMI classification after accounting for age and sex (Supplementary Table S2).

Table S2

| SNP | BMI Category | p-value |
| --- | --- | --- |
| rs10246939 | Overall | 0.74 |
| rs1726866 | Overall | 0.82 |
| rs713598 | Overall | 0.61 |
| rs35744813 | Overall | 0.48 |
| rs35874116 | Overall | 0.39 |
| rs838133 | Overall | 0.55 |

Supplementary Table S3.

| GENE | SNP | Nuc. Substitution* | AA substitution | Effect Allele** | MAF*** |
| --- | --- | --- | --- | --- | --- |
| TAS2R38 | rs713598 | C>G | Ala49Pro | G (Pro) | 0.42 |
| TAS2R38 | rs1726866 | G>A | Ala262Val | G (Ala) | 0.48 |
| TAS2R38 | rs10246939 | T>C | Ile296Val | C (Val) | 0.46 |
| TAS1R2 | rs35874116 | T>C | Ile191Val | C (Val) | 0.32 |
| TAS1R3 | rs35744813 | T>C | Intronic | T | 0.20 |
| FGF21 | rs838133 | A>G | Synonymous | A | 0.40 |

*All alleles are presented in the forward orientation

**Effect Allele: The allele associated with an increased preference for sweet taste.

***MAF: Minor Allele Frequency. Frequencies are obtained from dbSNP database. The minor allele corresponds to the effect allele in this study.

AA: Amino acid

Nuc: Nucleotide
